# Target heart rate to determine the normal value of coronary flow reserve during dobutamine stress echocardiography

**DOI:** 10.1186/1476-7120-9-10

**Published:** 2011-04-04

**Authors:** Ezequiel H Forte, Maria G Rousse, Jorge A Lowenstein

**Affiliations:** 1 Investigaciones Médicas. Buenos Aires. Argentina

## Abstract

**Background:**

The determination of coronary flow reserve (CFR) is an essential concept at the moment of decision-making in ischemic heart disease. There are several direct and indirect tests to evaluate this parameter. In this sense, dobutamine stress echocardiography is one of the pharmacological method most commonly used worldwide. It has been previously demonstrated that CFR can be determined by this technique. Despite our wide experience with dobutamine stress echocardiography, we ignored the necessary heart rate to consider sufficient the test for the analysis of CFR. For this reason, our main goal was to determine the velocity of coronary flow in each stage of dobutamine stress echocardiography and the heart rate value necessary to double the baseline values of coronary flow velocity in the territory of the left anterior descending (LAD) coronary artery.

**Methods:**

A total of 33 consecutive patients were analyzed. The patients included had low risk for coronary artery disease. All the participants underwent dobutamine stress echocardiography and coronary artery flow velocity was evaluated in the distal segment of LAD coronary artery using transthoracic color-Doppler echocardiography.

**Results:**

The feasibility of determining CFR in the territory of the LAD during dobutamine stress echocardiography was high: 31/33 patients (94%). Mean CFR was 2.67 at de end of dobutamine test.

There was an excellent concordance between delta HR (difference between baseline HR and maximum HR) and the increase in the CFR (correlation coefficient 0.84). In this sense, we found that when HR increased by 50 beats, CFR was ≥ 2 (CI 93-99.2%). In addition, 96.4% of patients reached a CFR ≥ 2 (IC 91.1 - 99%) at 75% of their predicted maximum heart rate.

**Conclusions:**

We found that the feasibility of dobutamine stress echocardiography to determine CFR in the territory of the LAD coronary artery was high. In this study, it was necessary to achieve a difference of 50 bpm from baseline HR or at least 75% of the maximum predicted heart rate to consider sufficient the test for the analysis of CFR.

## Background

Coronary atherosclerosis is a highly prevalent disease in our population, and not just the expression of an anatomic condition [1]. The functional status of this affection should be thoroughly analyzed. The valuable information provided by coronary angiography is not enough to study the functional status of atherosclerotic disease [2]. In these sense, several studies have demonstrated that management of coronary artery disease based on coronary artery anatomy without considering a functional approach is not superior to medical treatment [3]. Determination of coronary flow reserve (CFR) has been validated and supported by several trials studying coronary artery function [4]. In patients with known or suspected CAD stress echocardiography by semi-simultaneous analysis of wall motion and CFR provides critical diagnostic and prognostic information [4,6].

The use of dobutamine stress test as an alternative to exercise testing started in 1984 [7] and its indication has increased exponentially during the last 10 years [8]. Nowadays, dobutamine stress echocardiography is one of the pharmacological tests most used in stress-echo laboratories around the world [9]. The assessment of CFR using dobutamine stress echocardiography has been studied during the last years [10,11]. In this sense, CFR measured by this test has been compared to CFR measured by adenosine stress echocardiography, (adenosine is considered the best vasodilator stimulus to determine CFR), and has been validated in comparative studies [11]. After reviewing the published literature, and as we did not find any parameter indicating when coronary flow reserve should be measured during dobutamine stress echocardiography, we decided to conduct out own investigation. According to previous studies and to our personal experience, coronary flow velocity has a close relation with heart rate; thus, we focused on analyzing coronary blood flow velocity and CFR in each stage of dobutamine stress echocardiography in order to determine:

1- The target HR for each patient to reach a CFR ≥ 2 (considered the adequate limit to rule out significant coronary stenosis in the territory of the LAD coronary artery by the international literature and our own experience) [11-13] and, with this information, to determine the delta HR and the percentage of maximum predicted heart rate that should be reached to make another determination of CFR.

2- The feasibility of the test.

## Objetives

The goal of the present study was to determine at which value of heart rate, CFR should be measured during stress echocardiography. Thus, we evaluated the velocity in the LAD coronary artery in each stage of dobutamine stress echocardiography using transthoracic Doppler echocardiography, in order to find:

1- The delta heart rate and the percentage of maximum predicted heart rate that should be reached to determine CFR during dobutamine stress echocardiography.

2- The feasibility of estimating CFR during dobutamine stress echocardiography.

## Methods

### Study population

We prospectively included 33 patients without history of heart disease and low pre-test probability of coronary artery disease, who were referred to the echo-laboratory with indication of dobutamine stress echocardiography. Patients at high risk of coronary artery disease, history of ischemic heart disease, moderate to severe valvular heart disease, cardiomyopathy, left ventricular dysfunction and regional wall motion abnormalities were excluded. Also, cases with positive test results or CFR that did not reach the normal predetermined value (≥ 2) were not included in the analysis. Beta blockers were withdrawn 24 hours before the study. All participants gave informed consent before examinations. Table 1 describes the general characteristics of the population.

**Table 1:** Basal characteristics of the population

Patients	Total	%
Mean age	62.1 ± 9.2 years	
Men	12	36%
Diabetes	6	18%
Current smoking	7	21%
Hypertension	19	57%
Dyslipemia	10	30%
Reasons for ordering the study:		
Chest pain	12	36%
Preoperative	4	12%
Diagnosis of coronary artery disease	9	27%
Other	8	24%

### Methodology of study of the LAD coronary artery

The echocardiographic procedure was performed using two ultrasound systems, an ATL HDI-5000 Ultrasound System with a frequency of 4-7 MHz (Doppler frequency: 4 MHz) and a 2- 4 MHz matrix array transducer (Vivid 7, GE).

In color Doppler flow mapping, velocity range was set at an average Nyquist limit of 19.2 cm/s and, when heart rate increased, the Nyquist scale was adjusted at higher limit for a better visualization of the LAD flow.

The color gain was adjusted to provide optimal images, setting the color-write priority threshold above the tissue grey-scale gain in order to identify color blood flow in the LAD coronary artery. The anatomical position of the LAD running along the interventricular groove, a few centimeters beneath the surface of the skin, provides an excellent opportunity for its visualization and study.

With the patient in the left lateral decubitus position, the left ventricle was imaged in the low parasternal long-axis section, and the ultrasound beam was then tilted clockwise until the right ventricle disappeared and the interventricular groove was visualized with the mid-portion of the LAD. The distal LAD can be explored from the modified apical 3-chamber view.

The LAD coronary artery was visualized as a tubular red structure 2-4 mm long along the anterior interventricular groove, with a flow directed towards the apex (and transducer), generating a Doppler waveform above the baseline. Flow velocity was measured using pulsed Doppler echocardiography with a 2-3 mm sample volume wide, generating a typical biphasic pattern, with a smaller systolic component and a larger diastolic one.

The long axis sections were carefully adjusted to minimize the angle between the Doppler beam and the LAD flow; we did not perform angle correction to calculate CRF, as the potential error remains constant in the numerator and denominator of the equation, without changing the final results. We measured the flow velocity from the same position at each stage of dobutamine stress echocardiography.

Coronary flow reserve was calculated as the ratio between peak diastolic flow velocity and baseline diastolic flow velocity; final values of flow velocity represented an average of 3 cardiac cycles.

### Statistical Analysis

Continuous variables were expressed as mean and standard deviation and categorical variables as percentage. The association between variables was evaluated using Pearson's correlation coefficient. All calculations were performed using MedCalc statistical software.

### Study Protocol

**Figure 1:**
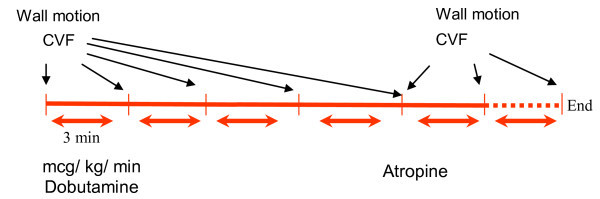
**Study protocol**. CFV: Coronary Flow Velocity.

All patients underwent complete color Doppler echocardiography. Digital images were obtained for wall motion analysis following conventional dobutamine stress echocardiography protocol. Diastolic velocities in the LAD coronary artery were measured during each stage. The hemodynamic variables - heart rate and blood pressure - were monitored. Dobutamine was administered by infusion pump in all patients. We did not use any contrast enhancement agent. Dobutamine infusion started at 5mcg/kg/min and increased in 3-minute stages to 10, 20, 30, 40 and 50 mcg/kg/min or until target heart rate was attained. Intravenous atropine was given (0.5 mg) until a maximum of 1.5 mg if the patient did not reach 85% of maximum HR with 30 mcg of dobutamine (Figure 1).

**Figure 2:**
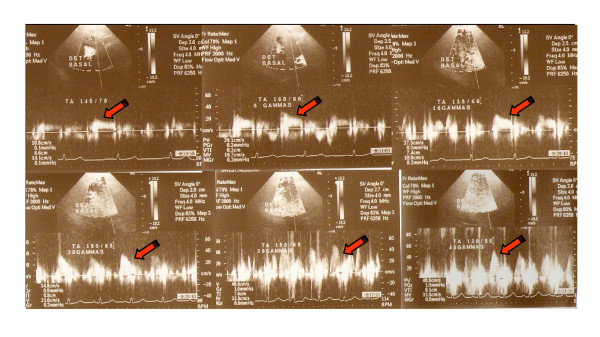
**Flow velocity in the left anterior descending coronary artery**. See the increase in peak velocity from 18.9 cm/s to 48.9 cm/s. CFR: 2.6.

The presence of symptoms and the hemodynamic variables such as heart rate, blood pressure and diastolic velocity in the LAD coronary artery were recorded in each stage (Figure 2).

CFR was calculated as the ratio between peak diastolic flow velocity and baseline diastolic flow velocity; final values of flow velocity represented an average of 3 cardiac cycles. As we have previously defined, abnormal CFR was considered with a value < 2 according to previous invasive and non-invasive studies [12,14].

## Results

### Feasibility

CFR could be determined in 31/33 patients (Feasibility 94%).

Among the 31 patients in whom CRF could be measured, only one did not reach the normal reference value (≥ 2)

### Complications

No major complications were reported. One patient presented supraventricular tachycardia that reverted spontaneously when the infusion was stopped.

### Hemodynamic variables

Mean arterial pressure (MAP) showed a small decrease due to the effect of the drug; the other variables did not present significant changes (Table 2). Intravenous atropine was administered in 10 patients (30%) and 16 (48%) needed 50 mcg/kg/min of dobutamine to complete the study.

**Table 2:** Hemodynamic variables and ratio of flow velocities

	VELOC (cm/seg)	HR (bpm)	Correlation with baseline flow in each stage	% increase	MAP (MmHg)	% HR increase
**Baseline**	24.93	64		0.00	94.4	
**5 MCG**	27.36	64.7	1.1	9.88	98.4	1.09375
**10 MCG**	31.1	69.6	1.3	24.90	98.9	8.75
**20 MCG**	38.9	81.2	1.5	56.22	99.5	26.875
**30 MCG**	46	99	1.8	84.74	93.3	54.6875
**40 MCG**	57.9	118	2.3	132.53	89.8	84,375
**50 MCG**	60.24	126.9	2.67	183.90	88.6	98.28125

### Wall motion analysis

Regional wall motion analysis was performed after digital image acquisition using a 16-segment model.

The test was negative for myocardial ischemia in all patients as wall motion improved in all the territories and systolic wall thickness increased.

### Analysis of CFR

**Figure 3:**
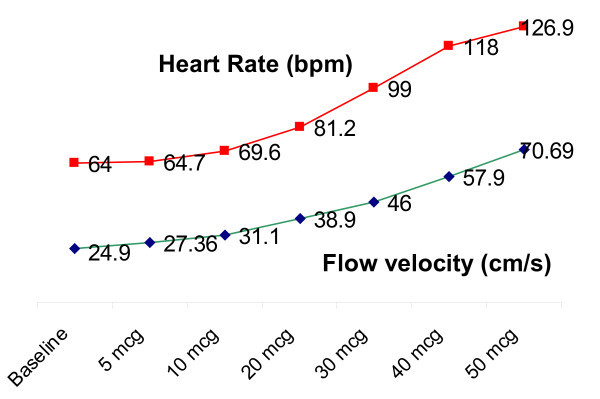
**Relation between HR and flow velocity in the left anterior descending coronary artery**.

Coronary flow velocity increased from a mean value of 24.9 ± 7.11 cm/s at baseline to a peak value of 69.24 ± 20.132 cm/s. The mean CFR at the end of the dobutamine test was 2.67 ± 0.62 (see table 2). A direct correlation was observed between heart rate and flow velocity in the LAD coronary artery with higher dose of dobutamine (Figure 3).

Only one 83 year-old male patient with a recent normal perfusion study and left ventricular hypertrophy in the echocardiogram did not reach a CFR ≥ 2.

Excluding this patient, we performed and independent analysis of the delta HR and the percentage of maximum predicted heart rate that should be reached to attain a CFR ≥ 2.

**Figure 4:**
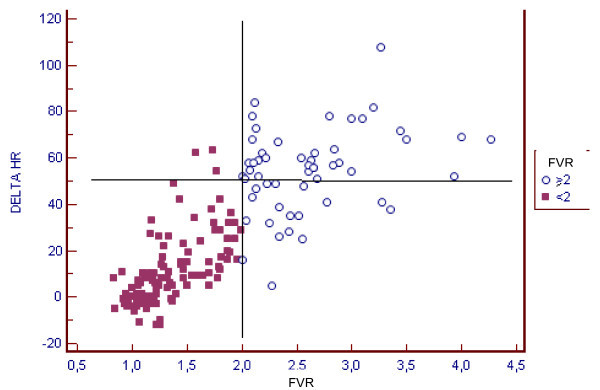
**Correlation between delta HR (max HR -baseline HR) and Flow Velocity Ratio (FVR) in each stage of the test**. R 0.8372 (95% CI 0.79-0.87) p < 0,001

We found an excellent correlation between delta HR (difference between baseline HR and maximum HR) and the increase in the CFR (correlation coefficient 0.84). In this sense, we found that when HR increased ≥ 50 bpm, CFR was ≥ 2 in 97.2% of patients (CI 93-99.2%; p < 0.001) (Figure 4).

**Figure 5:**
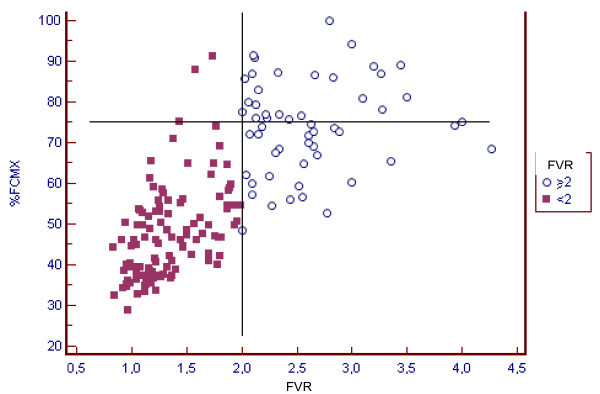
**Correlation between % of predicted heart rate and Flow Velocity Ratio (FVR) in each stage of the test**. R 0.73 (95% CI 0.65 - 0.79) p < 0,001

At the same time, we analyzed the percentage of maximum predicted heart rate necessary to attain a CFR ≥ 2, and we found a correlation coefficient of 0.73. In addition, 95.6% of patients reached 75% of their predicted maximum heart rate and attained a CFR ≥ 2 (CI 91.1 - 99%) (Figure 5).

Only two patients failed to reach these limits. When each patient was analyzed separately, one of them reached a CFR > 2 in the last stage with a HR of 149 bpm (delta HR: 68 bpm and percentage of maximum predicted HR: 91%) and HR decreased in other patient with dobutamine infusion of 30 mcg/kg/min; this patient required intravenous atropine and 50 mcg/kg/min of dobutamine to attain an adequate CFR.

## Discussion

The estimation of CFR using the method we have utilized is interesting due to its clinical and physiopathological value [15].

Despite dipyridamole is more suitable for the assessment of CFR [5,6], dobutamine is one of pharmacologic agents most commonly used during stress echocardiography [16]. However; the simultaneous estimation of CFR is not frequent in daily practice, with any of the above drugs; probably, this might be due to the lack of experience of echo-laboratories with this method, besides a relatively longer duration of the test that is not covered by health care programs.

Dobutamine exerts a complex effect on the coronary arteries: heart rate and myocardial contractility increase due to adrenergic stimulation, producing greater oxygen (O2) uptake and reactive hyperemia [17]. The direct effect of dobutamine produces vasodilatation by direct effect on epicardial coronary arteries and microcirculation (stimulation of β1, β2 and α adrenergic receptors in the vascular wall) [18,19].

Dobutamine hyperemia is equivalent to adenosine-induced vasodilatation in patients with ischemic response to dobutamine, but lower than that observed in non-ischemic patients. In normal patients, the effect of dobutamine on CFR is lower than that of adenosine according to studies using paired Doppler Flowires in the catheterization laboratory [20,21].

In addition, dobutamine increases contractility and myocardial O2 consumption, and produces release of vasodilatory substances such as adenosine, which act on epicardial arteries and microvasculature. Finally, dobutamine, by increasing the heart rate, induces flow mediated epicardial vasodilation [22,23].

The analysis of CFR has been studied in different clinical scenarios; however there is little information about coronary flow in each stage.

In a study performed at the Mayo Clinic by P. Pellikka et al., the feasibility of assessing the CFR was 97% from peak diastolic velocity. The authors concluded that CFR assessment during dobutamine stress echocardiography correlated well with wall thickening and detected ischemia early before development of wall motion abnormality, emphasizing the importance of determining CFR in this type of study [24].

We have not found any study analyzing and measuring the relation between heart rate and coronary flow reserve during this test.

In an excellent study, P. Meimoun et al. assessed CFR during dobutamine stress echocardiography and compared it to the CFR obtained with adenosine in the same group of patients and found a good correlation and concordance between the tests, in a wide range of LAD disease. Although a clear cut-off of heart rate was not established in that study, it was emphasized along the manuscript that a maximal achievable target heart rate was necessary during dobutamine infusion to obtain the good correlation and concordance between CFR-dobutamine and CFR-adenosine. Furthermore, a significant correlation was found in that study between CFR during dobutamine and change of rate-pressure product in patients with abnormal results [11].

In addition, the normal and abnormal values of CFR during dobutamine stress were clearly described in that study. A CFR < 2 with dobutamine stress echocardiography was found in all patients who had a positive test in the LAD territory (n = 8) and interestingly the same low CFR < 2 was obtained with adenosine in these patients [11].

Takeuchi et al. [10] studied 129 non selected patients. In patients without wall motion abnormalities, mean CFR was 2.76, similar to our findings. Coronary flow velocity was evaluated in each stage, yet changes in heart rate were not investigated.

It should be noted that the feasibility of analyzing CFR during dobutamine stress (around 90%) according to our previous experience and the results of previously mentioned studies [10,11] is a little lower compared to CFR obtained using vasodilator agents (about 90-95%) [25]. This might be explained by bias in patients selection as well as to the fact that, compared to dipyridamole, dobutamine increases heart rate, myocardial contractility and movements of the heart with displacement of the LAD; for these reasons, the estimation of CFR may be more difficult.

Finally, 97% patients achieved an adequate CFR with a delta HR of 50 bpm from the baseline stage. In the same sense, 95.6% of patients reached a normal CFR with 75% of their predicted maximum heart rate.

This study puts in evidence that it is technically recommended to obtain coronary artery flow in baseline conditions and after reaching the predicted heart rate (50 bpm greater than baseline HR or 75% of maximum predicted HR) without forgetting about wall motion response.

## Clinical applications

The importance of this study is that there is no need to measure CFR at each stage of the test; it can be determined at baseline and during high-dose of dobutamine infusion if HR is 50 bpm greater compared to baseline or has reached 75% of the maximum value predicted.

Further investigations are necessary to confirm the added value of estimating CFR using dobutamine stress echocardiography. Possibly, it may provide additional information regarding mid- and long-term prognosis besides increasing the sensitivity of the test as it has been already demonstrated with dipyridamole [6,26].

## Study limitations

The feasibility of the procedure might have been overestimated due to the relatively low number of patients included and to the selection of patients with low pre-test probability of coronary artery disease. However, as this is a physiopathological study, the hemodynamic data of the relation between drug dose, heart rate attained and CFR are enough to draw conclusions.

CFR was only evaluated in the territory of the LAD coronary artery as the feasibility of obtaining flow velocities from the other coronary arteries is low due to increased contractility secondary to dobutamine infusion.

As we did not perform coronary angiography in any patient, some doubts may arise regarding the absence of coronary artery lesions; yet the post-test probability was enough to rule them out.

It should be taken into account that CFR may be abnormal not only in lesions of the epicardial coronary arteries but also in several conditions that modify baseline coronary flow and its response to a stimulus. In this sense, the analysis of the result is complex [27,28]. However, it is widely accepted that a normal value (≥ 2) rules out a significant lesion in the territory evaluated [6,14,29].

The fact that only beta-blockers, and no other medical therapy, were withdrawn before dobutamine infusion could have had some hemodynamic influence [30].

We did not use contrast agents which could have improved the visualization of the LAD coronary artery [31].

## Conclusions

It is extremely important to reach a delta HR of at least 50 bpm or a HR of at least 75% of the maximum predicted heart rate to consider abnormal a CFR value.

The feasibility of determining CFR in the territory of the LAD during dobutamine stress echocardiography was high.

## Abbreviations and acronyms

CFR: Coronary Flow Reserve; HR: Heart Rate; LAD: Left Anterior Descending; MHz: Megahertz; MAP: Mean Arterial Pressure; O2: Oxygen.

## Competing interests

The authors declare that they have no competing interests.

## Authors' contributions

JAL has had the main idea and has participated in the making of the study. EHF has participated in the making of the study and drafted the manuscript. GR has performed the statistical analysis. All authors have participated in the discussion and correction.

All authors read and approved the final manuscript.
